# Drug induced toxic epidermal necrolysis: two case reports

**DOI:** 10.4076/1757-1626-2-7765

**Published:** 2009-09-09

**Authors:** Syed Nurul Rasool Qadir, Naeem Raza, Fozi Qadir

**Affiliations:** 1 Department of Dermatology, Combined Military HospitalKohat-75200Pakistan; 2 Department of Dermatology, PAF Hospital FaisalKarachi-8Pakistan; 3 Department of Emergency Medicine, Military HospitalPeshawar Road, Rawalpindi-51000Pakistan

## Abstract

**Introduction:**

Among the various drug induced dermatological entities toxic epidermal necrolysis and Stevens-Johnson’s syndrome occupy a primary place in terms of mortality. Prompt recognition of these conditions, immediate drug withdrawal and institution of appropriate treatment plays a vital role in reducing mortality. Drugs are by far the most common cause of toxic epidermal necrolysis, in which large sheets of skin are lost from the body surface making redundant the barrier function of the skin, with its resultant complications. The use of systemic corticosteroids in the treatment of toxic epidermal necrolysis has always been controversial, some consider corticosteroids life-saving while others believe that they increase mortality.

**Case presentation:**

We describe two cases of drug-induced toxic epidermal necrolysis, a male and a female, both caucasoids of Pakistani origin, one treated without any steroids and the other with them, who made complete recovery without any major complications or sequelae.

**Conclusion:**

The administration of systemic corticosteriods did not cause any major changes in outcome in our cases.

## Introduction

Toxic epidermal necrolysis also known as Lyell’s syndrome is a rare but potentially life threatening condition. It is primarily a cutaneous reaction to various precipitating agents, characterized by wide spread erythema and detachment of the epidermis from the dermis. Toxic epidermal necrolysis occurs sporadically, more commonly in adults, with a mean age of 46.8 years. There is no sexual or racial predilection. It is fairly rare with an annual incidence of 1.2 cases per million. An increased incidence has been observed in HLA-B 12 individuals.

## Case presentation

### Case report 1

A 60-year-old Pakistani male of Caucasoid origin was admitted to the dermatology unit with the following history. He had been operated for cataract 05 days ago. Post-operatively Co-trimoxazole tablets were given. Forty eight hours after taking the tablets the patient developed a severe burning sensation all over the body. This was followed by peeling of sheets of skin over the back, buttocks, thighs and neck. He had had a similar reaction to tetracyclines 12 years ago.

**Figure 1.**
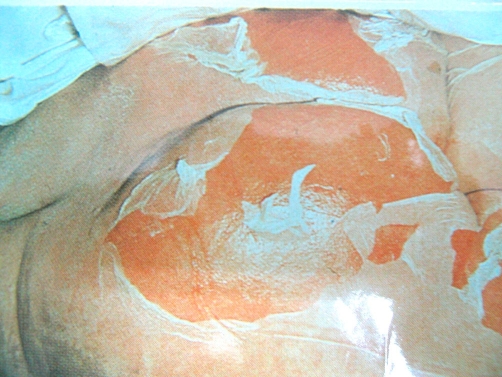
Peeling of sheets of skin and erosions over the buttocks (Case no. 1).

Initial examination revealed an asthenic, sick looking man with a B.P. of 130/90 mmHg, temperature 99 degree Fahrenheit and pulse 110/min. Hydration was poor. He was in obvious distress but conscious and oriented. On dermatological examination 40% of body surface area showed peeling of skin in sheets of more than 3 cm leaving behind superficial erosions. The back, buttocks, thighs and anterior aspect of the neck were primarily involved (Figure 1). Skin tenderness and Nickolsky’s sign were positive. The mucosae were not involved.

At the time of admission the bio-chemical profile was urea 9 mmol/lit, creatinine 274 umol/lit, potassium 3.6 mmol/lit, and sodium 137 mmol/lit. The serum total proteins were 4.9 mg/dl with an albumin level of 3.4 mg/dl and an albumin/globulin ratio of 1.5/1. The blood counts, urinalysis, electrocardiogram and chest roentogram were unremarkable. Skin biopsy showed a sub-epidermal split with necrosis of the epidermis and eosinophilic infiltration.

On the basis of body surface area involved a fluid requirement of 4 lit/24 hours was deduced, half of which was administered as Ringer’s lactate and the other half as glucose saline. He was also put on intravenous Ceftriaxone 1 gm twice daily. The urinary out-put improved and temperature returned to normal. Blood chemistry repeated on the 3^rd^ day revealed no abnormality. The very next day the patient developed multiple purpuric and ecchymotic spots over both thighs. Urgent investigations revealed raised fibrinogen degradation products levels in both urine and blood; however the serum fibrinogen, platelets and bleeding profile were normal. A diagnosis of compensated disseminated intravascular coagulation was made and fresh frozen plasma transfused. Over the next couple of days the skin lesions subsided and fibrinogen degradation products levels returned to normal. Subsequently the patient made an uneventful recovery and all the lesions healed without scarring.

### Case report 2

**Figure 2.**
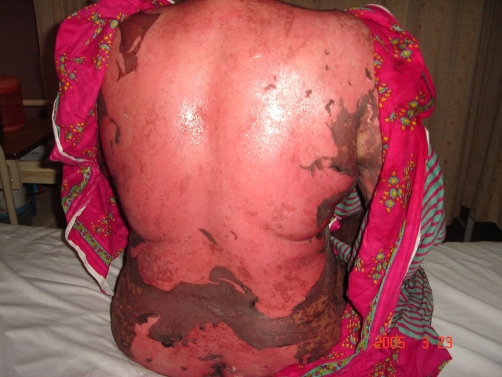
Peeled skin over the back and buttocks (Case no 2).

**Figure 3.**
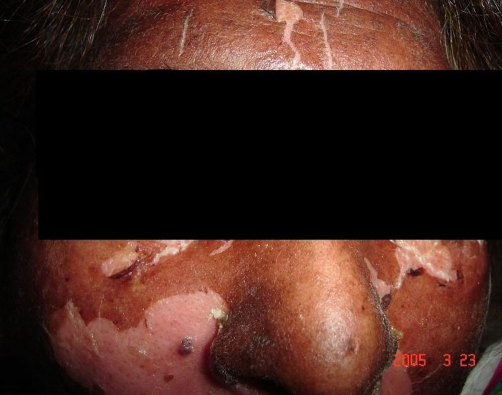
Involvement of the face (Case no. 2).

**Figure 4.**
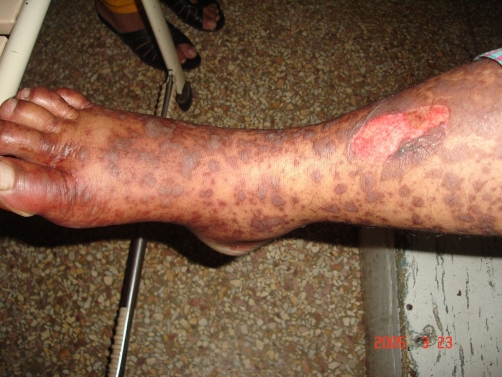
Rash and erosion over the lower limb (Case no. 2).

**Figure 5.**
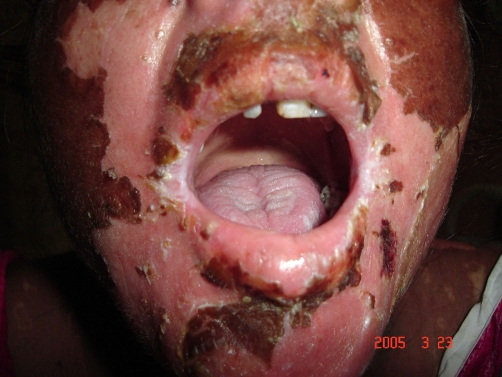
Involvement of the oral mucosa (Case no. 2).

**Figure 6.**
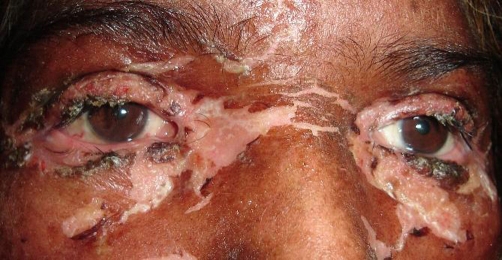
Involvement of the conjunctival mucosa (Case no. 2).

A 45-year-old Pakistani female of Caucasoid origin was admitted to the dermatology ward with extensive peeling of skin over the back and buttocks. She had taken a quinolone (moxifloxacin) for fever 05 days ago which was followed by a morbilliform eruption and widespread peeling of skin. She was a known diabetic for the past 10 years on insulin injections. On examination she was conscious but distressed, dehydrated and febrile with a temperature of 101 degree Fahrenheit, B.P. 130/90, pulse 98/min. She had skin peeling involving about 60% of the body surface area including the back, buttocks, face, neck and limbs (Figures 2-4). Oral and conjunctival mucosae were severely involved (Figures 5 and 6) and Nicholsky’s sign and skin tenderness were positive. Biochemical profile revealed raised serum urea 10.7 mmol/lit, creatinine 75 umol/lit, sodium 137 mmol/lit, potassium 3.8 mmol/lit, her ALT was also raised 87 IU but serum bilirubin and alkaline phosphatase were within normal limits. The blood counts, urinalysis, X-rays chest and electrocardiogram revealed no abnormality. Skin biopsy showed sub-epidermal clefting with a mixed infiltrate of polymorphs. The patient was treated with intravenous dexamethasone 3 mg 8 hourly and intravenous ceftriaxone 1 gm twice daily, intravenous insulin on a sliding scale along with topical antiseptic dressings and fluid and electrolyte replacement therapy. The steroids were gradually tapered off and the patient exhibited an uneventful recovery over the next 2 weeks; all the skin lesions healed without scarring and there were no sequelae except for a corneal opacity in the right eye.

## Discussion

Toxic epidermal necrolysis also known as Lyell’s syndrome was first described by Lyell in 1956. The word toxic alludes to the constitutional symptoms while necrolysis refers to the necrosis and detachment of the full thickness of the epidermis [1].

Drugs are the most common cause accounting for about 65%-80% of the cases. The most common offending agents are sulfonamides, NSAIDs, butazones and hydrantoins [2]. The risks related to different drugs are different even for closely chemically related products. Multiple precipitating causes may co-exist [3]. The earlier a causative agent is withdrawn the better is the prognosis. Patients on drugs with longer half-lives have an increased chance of dying [4]. An increased incidence of toxic epidermal necrolysis has been observed in patients with brain tumors, systemic lupus erythmatosus, acquired immune deficiency syndrome and high dose steroid therapy [5]. Other precipitating causes include viruses, bacteria, fungi, immunization, neoplasms, graft versus host disease, radiotherapy, beverages fumigants and idiopathic.

An immune mechanism is implicated in the pathogenesis but its nature is still unclear. It is primarily directed at drug modified epidermal cells [5]. Different immuno-inflammatory pathways with early participation of activated CD8 T-lymphocytes are involved. Blister fluid contains significantly higher levels of soluble interleukin-2 receptors (sIL-2R) probably related to a local down-regulation of an immune mediated cytotoxic reaction [6].

Microscopically, there is sub-epidermal bullae formation, with eosinophilic epidermal necrosis. The dermal vessels show endothelial swelling without any vasculitis or necrosis. Ultra-structurally there is damage to the basal and lower spinous levels of the epidermis and cleft formation at the lamina densa. Immuno-florescence is always negative [7].

The interval between the intake of a drug and the onset of symptoms generally ranges from a few hours to 22 days with a maximum of 38 days recorded in case of anti-tuberculosis drugs [8].

There is a prodormal phase in which there is burning sensation all over the skin and conjunctivae, along with skin tenderness, fever, malaise and arthralgias. This is followed by the eruptive phase in which a morbilliform rash erupts in the axillae and groins and rapidly becomes confluent into a pale, livid, confluent erythema, sparing only the hairy areas. In the acme or peak phase, vesicles and flaccid bullae appear which rupture quickly to leave denuded areas mainly at pressure points. Fluid and protein loss occurs as in 2^nd^ degree burns. Nickolsky’s sign is positive in erythematous skin.

As a rule oral, genital and anal mucous membranes are severely involved. Nail shedding and hair loss may occur. Mucous membrane involvement occurs in 85%-95% cases and precedes the skin involvement in one third of cases [5].

The following diagnostic criteria must be fulfilled for a case to be labeled as toxic epidermal necrolysis.

1. Bullae or erosions involving more than 20% of body surface area or three different anatomical sites.
2. Skin peeling in sheets of more than 3 cm.
3. Involvement of non-exposed skin.
4. Mucous membranes frequently involved.
5. Skin tenderness within 48 hours of rash.
6. Biopsy confirmation within 48 hours.
7. Fever.
8. Bullae arising on an erythematous background.
9. Exclusion of Staphylococcal scalded skin syndrome.

Investigations usually show leukocytosis, albuminuria, water and electrolyte imbalance and raised transaminases. Leukopenia and thrombocytopenia occur in some cases. Lymphopenia with selective depression of CD4 T-helper cells may occur.

Complications include hypovolemic shock, pulmonary edema, acute tubular necrosis, membranous glomerulonephritis, gastrointestinal hemorrhage, bronchopneumonia and disseminated intravascular coagulation. Cicatricial alopecia, anonychia, ectropion, entropion and corneal opacities can also occur.

Recovery is slow over a period of 14-28 days and relapses are frequent. There is a tendency for scarring in all but the mildest of cases. Mortality is 25%-50% and rises with age, being more than 50% above 60 years of age. Half the deaths occur due to secondary infection. Pulmonary edema, pulmonary embolism and gastrointestinal hemorrhage are other important causes of mortality. Sequelae occur in 40% of cases and are mainly ocular such as ectropion, entropion, synechieae, pannus formation and sicca syndrome. Reticulate skin pigmentation may occur over the affected areas [5].

Treatment is mainly supportive with removal of the precipitating agent, good nursing care preferably on a ripple bed, care of the eyes and mouth to prevent scarring and infection and maintenance of fluid and electrolyte balance. Intravenous fluids given during the first 24 hours should include 1 ml/kg/% of body surface area involved of macromolecules (albumin), plus 0.7 ml/kg/% surface area of isotonic saline. In addition 1500 ml of fluids should be given through the naso-gastric tube during the first 24 hours. Intravenous fluids should be supplemented with potassium.

The patient should be put on a high protein diet 2-3 gms/kg daily. At least 2000 cal should be given during the first 24 hours increasing by 500 cal/day upto a maximum of 4000 cal/day. Naso-gastric feeding is preferable in severely ill patients. Temperature should be maintained at 30-32 degree Celsius. Topical Chlorhexidine or Silver nitrate may be used to wash denuded areas. Silver sulfadiazine should be avoided due to the implications of sulfonamides in toxic epidermal necrolysis [9].

Anti-coagulation with Heparin for the duration of the hospital stay has been advocated [5]. Some authorities advocate immediate covering of denuded areas with biologic dressings like porcine xenografts, cryopreserved allografts or amnion and collagen based skin substitutes [10].

The role of steroids is controversial. Their efficacy has never been conclusively demonstrated. A number of cases of toxic epidermal necrolysis have occurred in patients who were already on high-dose steroids [11]. Retrospective studies indicate a higher death rate in patients on steroids and that steroids do not prevent the occurrence or extension of epidermal necrolysis [12]. Another study has reported 66% survival rate in patients managed without steroids as compared to a 33% survival in patients on steroid therapy. The former group had a decreased incidence of gastrointestinal ulceration, candidal infection and better recovery rate from septic complications [13].

Early infusion of high-dose Intravenous Immunoglobulins (IVIG) causes a rapid cessation of skin and mucosal detachment in a majority of patients [7]. According to a study patients treated with IVIG have 83% less chance of dying than those not treated with it [14]. Contrarily another study has shown no beneficial effect of IVIG therapy [15].

## Abbreviations

HLA: human leukocyte antigen
IVIG: intravenous immunoglobulins
NSAIDs: non steroidal anti inflammatory drugs
